# Accurate quantification of supercoiled DNA by digital PCR

**DOI:** 10.1038/srep24230

**Published:** 2016-04-11

**Authors:** Lianhua Dong, Hee-Bong Yoo, Jing Wang, Sang-Ryoul Park

**Affiliations:** 1 National Institute of Metrology, Beijing, 100013, P.R. China; 2 Korea Research Institute of Standards and Science, Daejeon, Korea; 3 University of Science and Technology, Daejeon, Korea

## Abstract

Digital PCR (dPCR) as an enumeration-based quantification method is capable of quantifying the DNA copy number without the help of standards. However, it can generate false results when the PCR conditions are not optimized. A recent international comparison (CCQM P154) showed that most laboratories significantly underestimated the concentration of supercoiled plasmid DNA by dPCR. Mostly, supercoiled DNAs are linearized before dPCR to avoid such underestimations. The present study was conducted to overcome this problem. In the bilateral comparison, the National Institute of Metrology, China (NIM) optimized and applied dPCR for supercoiled DNA determination, whereas Korea Research Institute of Standards and Science (KRISS) prepared the unknown samples and quantified them by flow cytometry. In this study, several factors like selection of the PCR master mix, the fluorescent label, and the position of the primers were evaluated for quantifying supercoiled DNA by dPCR. This work confirmed that a 16S PCR master mix avoided poor amplification of the supercoiled DNA, whereas HEX labels on dPCR probe resulted in robust amplification curves. Optimizing the dPCR assay based on these two observations resulted in accurate quantification of supercoiled DNA without preanalytical linearization. This result was validated in close agreement (101~113%) with the result from flow cytometry.

Accurate DNA concentration measurement that is traceable to the International System of Units (SI) is very important for producing reproducible measurements in many applications involving DNA analysis. Like many other analytical measurements, accuracy of DNA quantification requires application of high quality reference materials that are certified for its DNA concentration. So far, only a few certified DNA reference materials are available. Enumeration-based quantification does not require such calibration standards, which is of great importance when reliable DNA reference materials are not readily available. In addition, enumeration-based quantification can be utilized to quantify DNA reference materials due to its high precision and theoretical accuracy^1^,^2^,^3^,^4^.

One method of enumeration-based DNA quantification is directly counting the individual DNA molecule by flow cytometry, where the DNA molecules are fluorescently labeled and individually counted in a uniform flow stream^4^,^5^. The flow cytometric counting of individual DNA fragments for bacterial identification was first reported by a research team at Los Alamos National Laboratory^6^,^7^. Thereafter, several research teams reported the use of flow cytometric counting for DNA quantification^8^,^9^. In 2009, Korea Research Institute of Standards and Science (KRISS) was the first to report a reference method for systematic investigation of the analytical performance of DNA count-based quantification. Recently, Yoo *et al*.^4^ successfully improved the performance of their count-based instrument while working with plasmid DNA. The flow cytometric counting technique is unable to distinguish similar size DNA molecules with different sequences, and is therefore only suitable for quantifying an extremely purified DNA sample or a mixture of substantially different sizes.

Another approach for enumeration-based quantification is digital PCR (dPCR), which can provide absolute DNA copy number concentration^10^,^11^. The dPCR has a unique ability to measure low concentrations of specific DNA sequences, without the need for external calibrators in a complex background. Originally described in the 1990s, dPCR has since been increasingly used for DNA quantification^12^,^13^,^14^,^15^. Typically, dPCR involves partitioning a DNA sample into a large number of individual partitions or droplets prior to DNA amplification, following which, the number of partitions that contain amplified DNA is assigned to the positive partitions. A count of positive partitions/droplets gives a direct measure of the copy number concentration of the target DNA based on Poisson statistics^16^,^17^. The partitions can be produced by micro-well structures onto a chip^16^ or from aqueous droplets produced in an oil medium (droplet dPCR, referred as ddPCR)^18^,^19^.

dPCR has been used for quantification of linear DNA vector^2^ as well as genomic DNA^20^. However, dPCR can significantly underestimate the concentration of supercoiled DNA due to unsuccessful amplification or amplification delay^3^,^10^. One way to quantify the supercoiled DNA by dPCR is to digest the supercoiled DNA into a linear form^2^,^3^, but the quantification of supercoils is necessary in many applications. Non-viral supercoiled plasmid DNA are presently the most widely used vectors in the area of gene therapeutics and DNA vaccination due to their high efficiency of transfection and expression^21^,^22^. Pillai *et al*.^22^ reported that supercoiled vaccine DNA measurements provided a more accurate assessment of the potential to prime a CD8 response than the biological tests for its expression in transiently transfected cells^22^. Additionally, the US Food and Drug Administration (FDA) and China food and Drug Administration (CFDA) recommends that the proportion of supercoiled plasmid in the vaccines should be at least 80% and 90%, respectively^23^,^24^. This requirement is based on an understanding that the content of supercoiled DNA is a key indicator of plasmid quality, and that the supercoiled plasmid has superior biological activity as compared to other plasmid forms^25^. Therefore, all of these tests could benefit greatly from a more accurate and reliable enumeration-based dPCR measurement that does not require preanalytical linearization.

In the past decade, there have been a number of research reports on the performance characteristics of dPCR and flow cytometric counting. In order to broadly accept this as a reference analytical procedure, their performances need to be fairly assessed through an inter-laboratory comparison. Thus, piloted by the KRISS, the International Bureau of Weights and Measures (BIPM) coordinated an international comparison of CCQM P154 “Absolute quantification of DNA”, which aims to address the comparability of enumeration-based quantification method including dPCR and direct counting^26^. The CCQM P154 comparison report showed that enumeration-based analytical methods were highly capable in absolute quantification of low copy DNA concentration. In contrast, the dPCR can generate false results unless the PCR assay was well designed and the conditions were carefully optimized. Moreover, most of the laboratories significantly underestimated the concentration of the supercoiled plasmid DNA by dPCR, which suggests that some factors need to be considered when using dPCR for quantifying supercoiled DNA.

A new bilateral comparison between KRISS and NIM was implemented for analyzing 3 different levels of supercoiled DNA sample. The objective of this study was to investigate the ability of accurate quantification of supercoiled DNA by dPCR, and to assess the comparability of supercoiled DNA quantification by dPCR and direct counting.

## Material and Methods

### DNA sample and PCR assay

Twelve vials containing 3 different levels of unknown concentrations of supercoiled pBR322 plasmid DNA in TE buffer (10 mM Tris-HCl, 1 mM EDTA, pH 8.0) were provided by KRISS. Each of the 4 vials contained the same copy concentration of DNA labeled as A for level 1, B for level 2 and C for level 3. Supercoiled pBR322 sample (labeled as O99) from CCQM P154 was also used to test the effect of plasmid conformation on dPCR quantification. Two PCR assays (Assay I and Assay II) were designed for the quantification of pBR322 plasmid using Primer Express 3.0.1 (Applied Biosystems). The sequence of the primer and probe pairs are listed in Table S1 of the supplement material.

Two additional plasmids, pNIM-001 and pNIM-002 in 1 × TE_0.01_ (10 mM Tris-HCl, 0.01 mM EDTA, pH = 8.0) containing maize line NK603 (108 bp) and BT11 (93 bp) event specific gene fragment, respectively, constructed by NIM were used to evaluate the effect of PCR master mix on qPCR performance. The PCR assays and reaction components for amplifying pNIM-001 and pNIM-002 are listed in the supplemental material (Table S2).

### Enzymatic digestion of the plasmid

The pBR322, pNIM-001, and pNIM-002 plasmids were linearized by *EcoR*1 (Takara) that does not target any sequence in the PCR amplicons. Enzymatic digestion mixture was comprised of 2 μL of 10 × buffer, 1 μL of *EcoR1* (15 U/μL) restriction enzyme, 10 μL of plasmid DNA, and 7 μL of ddH_2_O. Non-DNA and non-enzyme controls were prepared by replacing the DNA and enzyme with 10 μL of 1 × TE_0.1_ (10 mM Tris-HCl, 0.1 mM EDTA, pH = 8.0) and 1 μL of 1 × TE_0.1_ in the master mix, respectively. The enzymatic reaction was carried out for 1 h at 37 °C, and inactivated for 15 min at 65 °C. After the enzymatic reaction, the DNA was analyzed on the qPCR or dPCR instrument. Agarose gel analysis with fluorescent staining was used to check if the restriction digestion was completed, the electrophoresis image was in Fig. S1 (in supplemental material).

### Comparison of the PCR master mix for the PCR performance

To assess the impact of the PCR master mix selection on PCR performance of plasmid DNA with different conformations, 3 different plasmid DNA (pBR322, pNIM-001, and pNIM-002) with linearized or supercoiled conformation were analyzed on Roche480 real-time quantitative PCR machine (Roche, Sweden). Three PCR master mixes (abbreviations underlined): Gene Expression master mix (GE; Life Technologies), 16S DNA Free master mix (DF; Molzym) and Environmental master mix (EN, Life Technologies) were used. The reaction mixture preparation for each plasmid with 3 different master mixes is described in the supplemental material (Table S3). The PCR thermal profiles contain a 10 min activation period at 95 °C, followed by 45 cycles of a 2 steps thermal profile of 15 s at 95 °C denaturation and 60 s at 60 °C for combined annealing-extension.

### Quantification of unknown sample by dPCR

Digital PCR was quantified on the BioMark System (Fluidigm, San Francisco, CA) using 37 K qdPCR (48 × 770) integrated IFC controller (Fluidigm, San Francisco, CA). Three different vials from each level of unknown sample, labeled as A1, B1, and C1 were first selected for optimizing the DNA concentration on the 48 × 770 chip and for assay comparison. The reaction volume was prepared in 5 μL containing 2 × DF master mix, forward and reverse primer, probe, ROX, and 20 × GE loading (see Table S4 in the supplement material). To minimize the uncertainty from pipetting, the PCR reagents other than DNA template were premixed, and the final reaction mix was prepared gravimetrically by combining the DNA and PCR reagents. The PCR thermal profile was the same as that for the qPCR except for 50 cycles instead of 45 cycles. After optimization of the DNA concentration, the remaining 9 vials (three vials from each level) were then quantified by dPCR by using Assay I with HEX labeling. Each vial was analyzed in 5 replicates. The final stock concentration for each level of the sample was calculated by averaging the 15 measurements of 3 vials in total.

### Data Analysis

The stock concentration (*C*, copies/mg) of the 3 unknown samples was calculated using equation (1) and (2), where *M* is the number of copies per panel, *P* is the PCR positive partitions, *N* is the total partitions, *V*_*p*_ is the partition volume, *D* is the dilution factor used to dilute the DNA with the PCR master mixture, and *ρ* is the density of the PCR mixture with DNA. Statistical analysis was performed with the estimated copy number data (copies/mg) derived from the positive partitions by a 2 tailed t-test, where *p* value smaller than 0.05 (at the 95% confidence level) indicates a statistical significance. Grubbs tests were performed where necessary, and outliers were removed where *p* values were <0.00001.


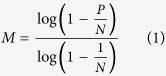



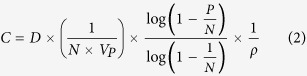


### Uncertainty evaluation

The uncertainty for *C* was related to the partition volume (*V*_*P*_), copy number per panel (*M*), density (*ρ*) and dilution factor (*D*), which was calculated using equations (3) and (4). The relative standard uncertainty of the partition volume 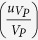, was determined through analysis of an individual partition volume. Factors such as the repeatability of the x, y, and z axis measurement, microscope calibration, and focus were all taken into account in the estimation of uncertainty. For the relative standard uncertainty of the density 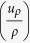, uncertainty sources like weighing repeatability, uncertainty of the balance and pipettes used in the density measurement were considered. The expanded uncertainty was calculated by multiplying the combined standard uncertainty by a coverage factor (*k* = 2). This provided a level of confidence of 95%.


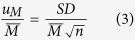



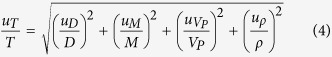


### Sample preparation and quantification by direct counting

The pBR322 plasmid DNA (Fermentas, #SD0041, Canada) was serially diluted in 1 × TE buffer thrice at 1/100-fold to result in 1/1,000,000 dilution. Finally, unknown samples were obtained by dividing the 1/1,000,000 diluted solution into 3 different concentration levels.

For direct counting, 300 μL of each unknown sample was gravimetrically added into 1.2 mL running buffer (10 mM Tris/HCl at pH 9.5 + 10% dimethyl sulfoxide) that contained 0.1 X SYBR Gold dye (S-11494; Invitrogen, Carlsbad, CA). For counting the sample, the well-mixed solution was introduced into the fluidic channel by applying 0.07 MPa for 60 seconds. The sample solution was replaced with the running buffer, and then 0.07 MPa with 20 kV was applied as the counting started. The direction of the electric field was set opposite to that of the hydrodynamic flow. Counting was performed for 2 minutes. For repeated counting, the fluidic channel was occasionally cleaned by flushing with 0.1 N NaOH. Each sample solution was thoroughly counted 3 times. The fluidic channel was prepared with a piece of 50 × 50 μm rectangular fused silica capillary (WWP050375, polymicro technologies) about 1 m in length. A detection window was projected ~60 cm away from the injection end by removing the protective coating. An in-house software program prepared using the Turbo-C language along with Microsoft Excel^TM^ and Microcal Origin^TM^ was utilized for signal processing and data reduction. More detailed experimental conditions are available in previously published reports^4^.

## Results and Discussion

### Optimization of dPCR conditions

**Figure 1:**
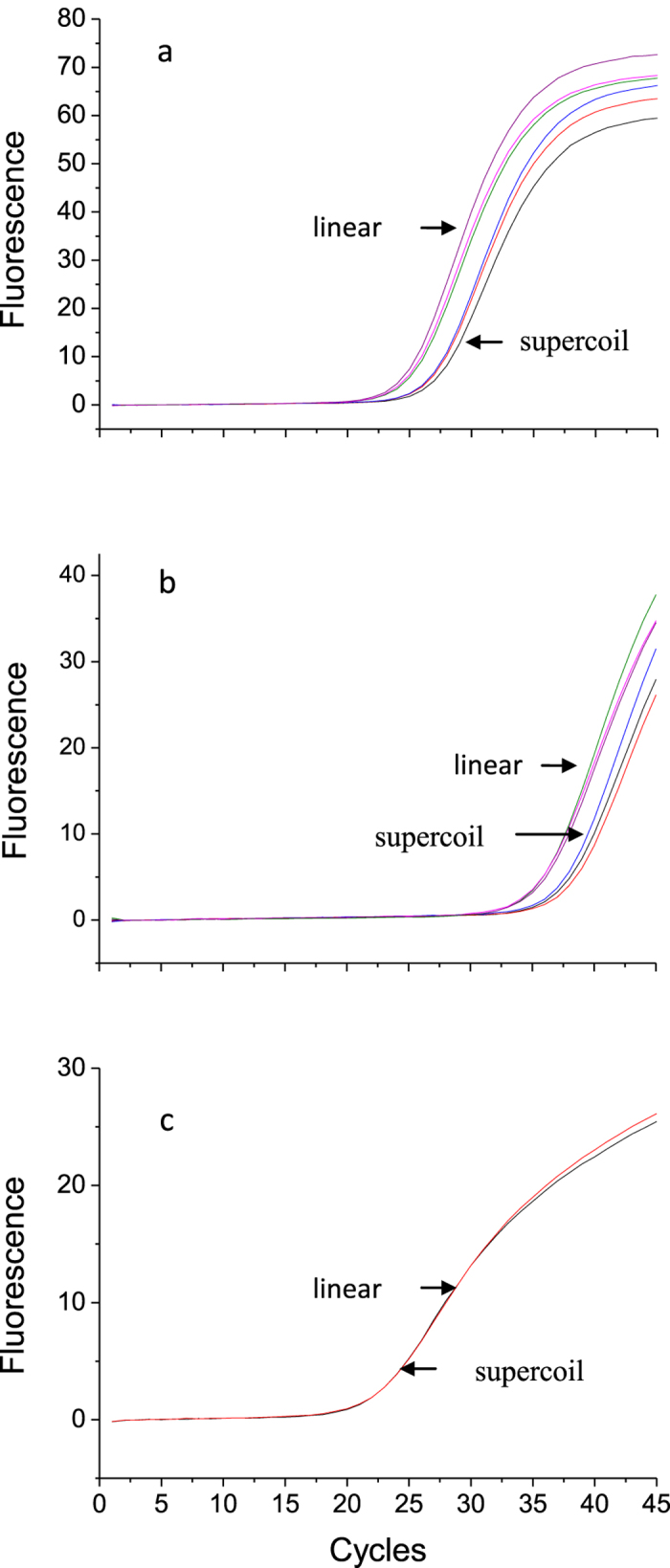
PCR amplification curves of linearized and supercoil pBR322 plasmid with (**a**) Gene Expression master mix (GE), (**b**) Environment master mix (EN) and (**c**) 16S DNA Free master mix (DF).

Among various dPCR conditions, the selection of a PCR master mix critically influenced the performance of dPCR, especially when the supercoiled DNA sample was used. As reported previously^20^, using different master mixes and templates can lead to significantly different results. In this study, 3 different PCR master mixes (GE, EN, and DF) were tested for pBR322 plasmid DNA in 2 different conformations (linearized and supercoiled). The qPCR amplification curves of pBR322 with other master mixes are compared in Fig. 1a–c. The amplification curves for the supercoiled form were greatly delayed compared to those for the linearized form with GE or EN mixes (Fig. 1a,b). The supercoiled structure of the template might not have completely melted to expose the sites for priming, probing, or extending in the environment created by the master mixes. Due to this, a large portion of the target templates fail to amplify in the beginning which causes an amplification delay. Contrary to this, there was no significant difference in the Cq values between supercoiled and linearized plasmid when using DF (Fig. 1c). We speculate that DF contains some ingredients that might help the supercoiled plasmid to fully untwist and expose the designated target regions.

To verify whether this observation is conformation-specific instead of sequence-specific, the master mixes were applied to qPCR of 2 other plasmid DNAs (pNIM-001 and pNIM-002), the results for which are compared in Figs S2 and S3 (supplemental material). For both plasmid DNAs, GE and EN showed substantial delay in the rise of the amplification curves for the supercoiled forms as well as for the supercoiled pBR322 DNA. For supercoiled forms of pNIM-001 and pNIM-002, no such amplification delays were observed with DF. Therefore, such delays are rather conformation-specific and profoundly dependent upon the formulation of a PCR master mix. We are unsure about the ingredients that make this remarkable difference, and they may be part of the manufacturer’s proprietary information. Nevertheless, this work strongly demonstrates a possibility of successfully amplifying the supercoiled DNA by using an appropriately formulated PCR master mix.

**Figure 2:**
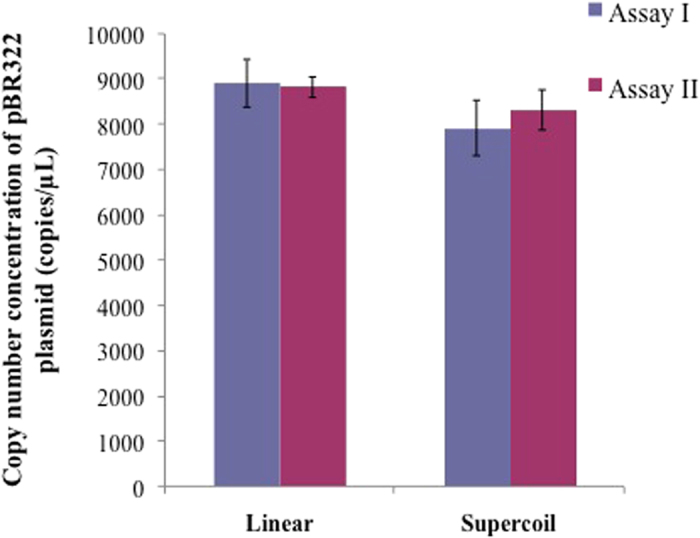
Comparison of digital PCR quantification with 16S DNA Free master mix (DF) for linearlized and supercoiled pBR322 plasmid sample (O99) using Assay I and Assay II (statistically insignificant for Linear and Supercoil DNA, *p* > 0.05).

Since DF showed indistinguishable amplification performance on a supercoiled and linearized DNA, it is likely that there would be no difference in dPCR quantification of supercoiled DNAs relative to their linearized forms. The dPCR quantification of supercoiled pBR322 and its corresponding linearized pBR322 plasmid using DF is shown in Fig. 2. The data demonstrated no significant difference in the results of dPCR quantification of supercoiled and linearized DNA samples. Therefore, preanalytical linearization of supercoiled DNA is not necessary. Direct dPCR quantification of supercoiled DNA avoids the source of extra uncertainty measurement arising from the process of enzymatic linearization. Three unknown samples from this study were then directly quantified by dPCR using DF.

On the other hand, we tried to optimize the PCR condition with GE by enhancing the annealing temperature and increasing the total number of PCR cycles. However, the results for supercoiled DNA was still underestimated (Fig. S4 in the supplemental material) compared with its corresponding linearized DNA, which indicates GE can not successfully quantify supercoiled plasmid by dPCR without pre-linearization. Furthermore, concentration of the linearized pBR322 quantified by using GE mix was statistically significant compared with that quantified by using DF mix. This finding is as same as that in CCQM P154 study for supercoiled DNA sample: most of participants underestimated the concentration of supercoiled DNA by dPCR with GE mix^26^. Additionally, a contamination was found in the GE mix. It has been reported that the a number of commercial PCR master mixes were contaminated with DNA that interfered with the measurement of pUC19 27, which is a reconstructive pBR322. This contamination was confirmed by the qPCR when using GE and EN mix. Therefore, it is impossible to accurately quantify pBR322 with GE and EN mix.

Appropriate selection of the fluorescent labels (FAM and HEX) also resulted in a substantial difference in the dPCR performance. Generally, FAM labels shows more intensive fluorescence signal compared to the HEX labels. Interestingly, high quality dPCR amplification curves were always obtained with HEX labels, whereas the opposite was observed with FAM labels (Figs S5 and S6 in the supplemental material). This was confirmed from the results of 48 replicates, each with 2 repeated measurements. Consequently, the quantitative result was very sensitive to the Cq threshold setting when using FAM labels. Especially for Assay II, a Cq threshold difference of only 0.01 led to a substantial change in the quantitative result (*P* = 0.0002) (Fig. S7 in the supplemental material). In contrast, the quantitative result was not sensitive to the Cq threshold of HEX labeling. There was no significant change in the obtained copy number concentrations even when the Cq threshold was increased from 0.05 to 0.10 (Fig. S8 in the supplemental material). Thus, HEX labeling was used for all the other subsequent assays.

**Figure 3:**
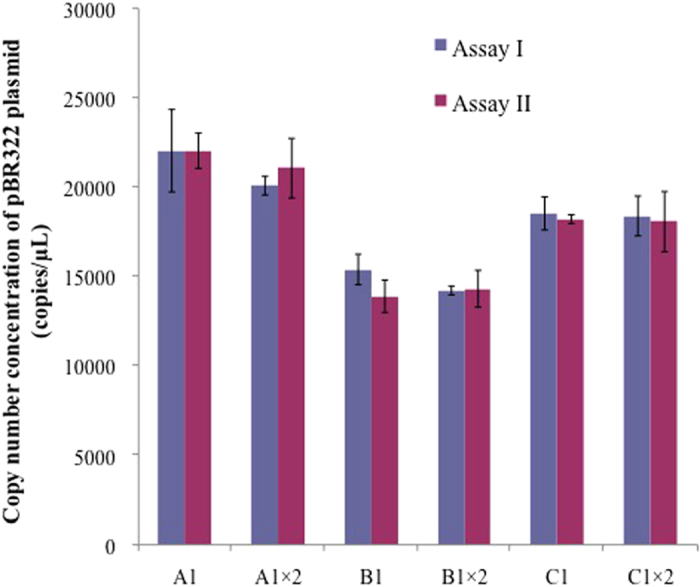
Supercoiled pBR322 plasmid DNA concentration optimization for digital PCR by using Assay I and Assay II labeling with HEX (A1 × 2, B1 × 2 and C1 × 2 are the two times dilution of sample A1, B1 and C1, statistically insignificant for sample A1, B1 and C1 between two assays and two dilutions, *p* > 0.05).

With the same fluorophore, no significant difference (*P* > 0.05) was observed between 2 assay setups using different primers and probe as shown in Fig. 3. It is likely that once the assay is well optimized, the sequence/position of the primers and probe is only a minor factor in the dPCR performance. In addition, different dilutions of the original samples did not lead to non-linearity in the quantification.

Two dilutions (no dilution and 2-fold dilution) of 3 levels of the sample (A, B, and C) were analyzed using 2 assays as shown in Fig. 3. T-test showed no significant difference in the quantification results between 2 dilutions of each sample (*P* > 0.05). This indicates that both the dilutions of the 3 samples were in the optimum concentration range for dPCR quantification. As indicated in Table S5 (supplemental material), positive counts for all the tested panels were in the range of 538 and 761, indicating that the final dilution was appropriate to avoid saturation. At the same time, substantially high positive counts for the most diluted samples helped to reduce the statistical uncertainty. These results indicate that the chosen dilution series were good for accurate dPCR quantification. To minimize the uncertainty source, no dilution was chosen for downstream quantifications.

The use of negative template controls (NTC) for dPCR quantification is often problematic, as they sometimes produce positive partitions most likely due to a master mix contaminated with various plasmid sequences. The reagents used in the present study did not lead to any positive partitions as seen in the hit map of NTC samples (panel 22 and 34 in Fig. S9).

### Digital PCR measurement results and uncertainty evaluation

**Figure 4:**
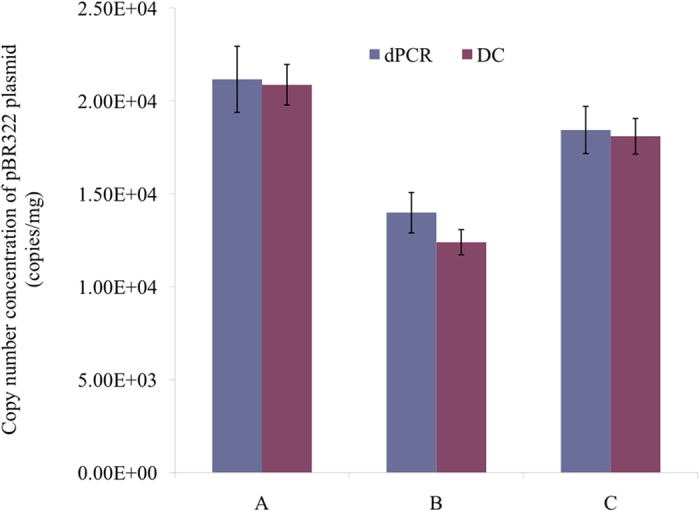
Measurement results of surpercoiled plasmid quantified by digital PCR (dPCR) and direct counting (DC) (statistical insignificance for sample (A–C) between two dPCR and DC, *p* > 0.05).

The original copy concentrations of the 3 unknown samples quantified by dPCR are shown in Fig. 4. The number of positive count and mean copies per partition for each technical replicates is listed in Table S6 (in the supplement material), and the result of the hit map is shown in Fig. S10. There were no positive partitions for NTC, indicating that the quantification results were not affected by contamination. The original concentrations for sample A, B and C were calculated to be 21,164, 14,550, and 18,435 copies/mg, respectively, with relative standard deviations (RSD) of 8.3%, 4.9%, and 6.4%, respectively. A large variation in sample A measurement repeatability was attributed to its relatively high mean copies per partition obtained from the original sample concentration, since precision becomes poor as the number of positive partitions approach saturation^28^. The uncertainty breakdown of the measurements for each sample is shown in Table 1. The precision uncertainty for 3 samples were between 48% and 78%, suggesting that the dPCR uncertainty could be greatly decreased if there is improvement in the measurement repeatability or precision. However, the precision depends on 2 factors, the mean number of molecules per partition^10^,^16^ and the number of partitions. dPCR can give most precise measurements at an optimal concentration of ~1.59 molecules per partition^29^, and the precision cannot be further improved with a fixed number of partitions. Therefore, an estimate of the concentration is necessary to obtain the most precise measurements, before applying the dPCR. On the other hand, the theoretical precision can be enhanced with an increase in the number of partitions. Due to our successful measurement and uncertainty evaluation of the partition volume of 48 × 770 digital chip, the expanded uncertainty (6.81 ~ 8.41%) was much lower than the previous reports using the same instrumentation^10^,^17^.

Using the Raman microscope (InVia, Renishaw), we obtained a slightly higher partition volume (0.872 nL) as opposed to the partition volume (0.844 nL) reported by the manufacturer. However, the partition volume of 0.872 nL was used to calculate all the original sample concentrations. The relative uncertainty of our own estimation of the partition volume was 1.47%.

### Limit of detection for the supercoiled DNA by dPCR

**Figure 5:**
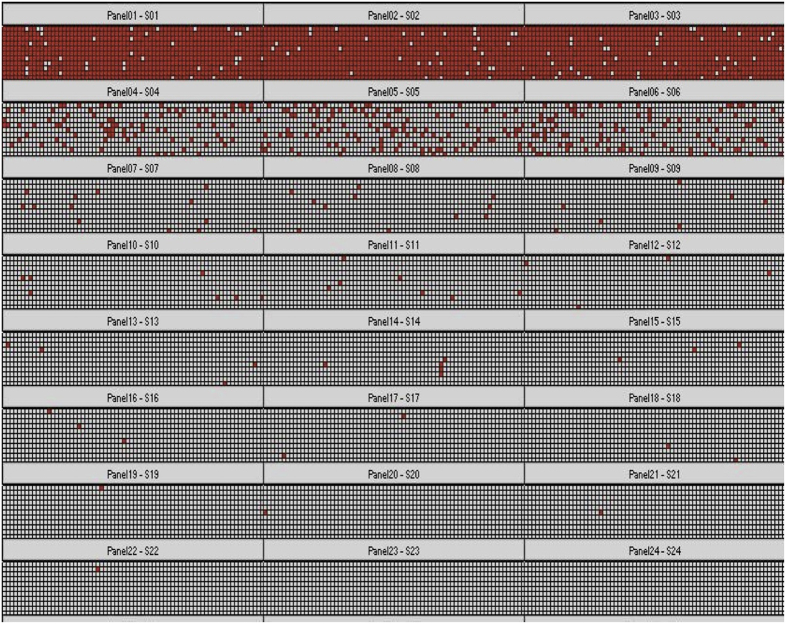
Digital PCR hit map of serial dilution of pBR322 (sample B) by using Assay I labeling with HEX (Panel 1–18, three replicates of each dilution from SD1 to SD6, panel 19–22, four replicates of dilution SD7, panel 23–24, NTC).

A serious dilution of sample B was prepared to determine the limit of detection of supercoiled DNA by dPCR. The supercoiled DNA concentration (copies/μL) in each dilution and the measured target copies per panel was shown in Table S7 (in the supplemental material). There was a linear relationship (R^2^ = 0.984, Fig. S11) between the nominal concentration and the measured concentration by dPCR. The dPCR hit map of each dilution (from SD1 to SD7) with replicates was shown in Fig. 5. It is interesting to note that a single positive partition was assigned in the four replicate panel (panel 19 to 22), when the nominal concentration was one copies per panel (corresponding to 8 copies/μL before diluted with PCR mixture in SD7, in Table S7), indicating that the limit of detection of supercoiled DNA is one copies per panel when using dPCR. The proposed dPCR method, thus can be used to detect low to 10 copies/μL of supercoiled DNA.

### Validation by comparison with direct counting analysis

The validity of the proposed dPCR quantification of supercoiled pBR322 DNA was ultimately tested by comparing the results with that of the flow cytometric directing counting. As shown in Fig. 4, a very close agreement of 101% and 102% for sample A and C, respectively, was obtained. Relatively higher difference in sample B is likely due to unsatisfactory repeatability of the methods. Nevertheless, these results are significant when considering that the supercoiled DNA quantification is typically underestimated by 30–50% by dPCR^4^. A close agreement with the results from the direct counting strongly suggests that PCR-induced artifact was almost completely avoided in the optimized dPCR assay. The direct counting does not include PCR amplification in its measurement procedure.

Another approach to evaluate a dPCR assay is to employ multiple assays using different primer pairs targeting the same template, and close agreement amongst those results would suggest the assay validity. In the present work, Assay I and Assay II were implemented for the purpose of mutual validation. However, there could still be PCR bias in those results. Cross validation using 2 methods with totally different measurement principles are rather compelling in method validation.

It is highly feasible to directly quantify supercoiled DNA by dPCR because it not only simplifies the measurement procedure, but also reduces the sources of measurement uncertainty. Furthermore, supercoiled DNA is more stable for long term storage, and can be used for successful dPCR.

## Conclusions

This study demonstrates that a substantial improvement in the commonly observed underestimation of the supercoiled DNA concentration can be obtained by dPCR, which also obviates the need for preanalytical linearization of supercoiled DNA. The linearization process introduces not only extra measurement uncertainty, but also renders the DNA susceptible to degradation. The validity of the improved dPCR quantification of supercoiled DNA was confirmed in close agreement with the results of the flow cytometric counting. Here, bilateral comparison of unknown samples was used to obtain a close agreement. Additionally, the results confirmed the previous findings that dPCR can easily leads to false results, especially with that of the supercoiled DNA. However, with careful optimization of dPCR assay conditions such as PCR master mixes and fluorescent labels, valid quantification results can be obtained even for supercoiled DNAs. The proposed dPCR assay can successfully detect low to 10 copies/μL of supercoiled DNA.

## Additional Information

**How to cite this article**: Dong, L. *et al*. Accurate quantification of supercoiled DNA by digital PCR. *Sci. Rep.* **6**, 24230; doi: 10.1038/srep24230 (2016).

## Supplementary Material

Supplementary Information

## Figures and Tables

**Table 1:** Uncertainty components for the three unknown samples.

**Sample**	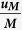 1 **(%)**	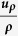 2** (%)**	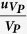 3** (%)**	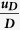 4** (%)**	**Combined uncertainty (*****u***_***c***_**) (%)**	**Expended uncertainty (*****U, k***** = 2) (%)**
A	3.73	1.07	1.47	0.68	4.20	8.41
B	2.26	1.67	1.47	0.68	3.24	6.49
C	2.81	1.03	1.47	0.68	3.41	6.81

^1^relative standard uncertainty of measurement repeatability.

^2^relative standard uncertainty of measurement of density.

^3^relative standard uncertainty of measurement of partition volume.

^4^relative standard uncertainty of dilution factor.

## References

[b1] Haynes R. J. . Standard reference material 2366 for measurement of human cytomegalovirus DNA. J. Mol. Diagn. 15, 177–185 (2013). 23321018 10.1016/j.jmoldx.2012.09.007

[b2] Corbisier P., Bhat S., Partis L., Xie V. R. & Emslie K. R. Absolute quantification of genetically modified MON810 maize (Zea mays L.) by digital polymerase chain reaction. Anal. Bioanal. Chem. 396, 2143–2150 (2010). 19816678 10.1007/s00216-009-3200-3

[b3] Dong L., Meng Y., Wang J. & Liu Y. Evaluation of droplet digital PCR on characterization of Plasmid Reference Material for Quantifying Ammonia Oxidizer and Denitrifier. Anal. Bioanal. Chem. Online 10.1007/s00216-013-7546-1, 1701–1712 (2014). 24493332 10.1007/s00216-013-7546-1 PMC3936116

[b4] Yoo H.-B. . A candidate reference method for quantification of low concentrations of plasmid DNA by exhaustive counting of single DNA molecules in a flow stream. Metrologia 51, 491–502 (2014).

[b5] Lim H.-M. . Count-based quantitation of trace level macro-DNA molecules. Metrologia 46, 375 (2009).

[b6] Kim Y. . Bacterial fingerprinting by flow cytometry: Bacterial species discrimination. Cytometry 36, 324–332 (1999). 10404148 10.1002/(sici)1097-0320(19990801)36:4<324::aid-cyto7>3.0.co;2-k

[b7] Ferris M. M. . Statistics of single-molecule measurements: applications in flow-cytometry sizing of DNA fragments. Cytometry A 60, 41–52 (2004). 15229856 10.1002/cyto.a.20000

[b8] Chao S.-Y., Ho Y.-P., Bailey V. & Wang T.-H. Quantification of Low Concentrations of DNA Using Single Molecule Detection and Velocity Measurement in a Microchannel. J. Fluoresc. 17, 767–774 (2007). 17653837 10.1007/s10895-007-0194-0

[b9] Zheng J. & Yeung E. S. Counting Single DNA Molecules in a Capillary with Radial Focusing. Australian J. Chem. 56, 149–153 (2003).

[b10] Bhat S., Herrmann J., Armishaw P., Corbisier P. & Emslie K. Single molecule detection in nanofluidic digital array enables accurate measurement of DNA copy number. Anal. Bioanal. Chem. 394, 457–467 (2009). 19288230 10.1007/s00216-009-2729-5

[b11] Bhat S. . Comparison of methods for accurate quantification of DNA mass concentration with traceability to the international system of units. Anal. Chem. 82, 7185–7192 (2010). 20690645 10.1021/ac100845m

[b12] Sykes P. J. . Quantitation of targets for PCR by use of limiting dilution. Biotechniques 13, 444–449 (1992). 1389177

[b13] Vogelstein B. & Kinzler K. Digital PCR. Proc. Natl. Acad. Sci. USA 96, 9236–9241 (1999). 10430926 10.1073/pnas.96.16.9236 PMC17763

[b14] Sanders R. . Evaluation of digital PCR for absolute DNA quantification. Anal. Chem. 83, 6474–6484 (2011). 21446772 10.1021/ac103230c

[b15] Lo Y. M. . Digital PCR for the molecular detection of fetal chromosomal aneuploidy. Proc. Natl. Acad. Sci. USA 104, 13116–13121 (2007). 17664418 10.1073/pnas.0705765104 PMC1934923

[b16] Dube S., Qin J. & Ramakrishnan R. Mathematical Analysis of Copy Number Variation in a DNA Sample Using Digital PCR on a Nanofluidic Device. Plos One 3, e2876 (2008). 18682853 10.1371/journal.pone.0002876 PMC2483940

[b17] Pinheiro L. B. . Evaluation of a Droplet Digital Polymerase Chain Reaction Format for DNA Copy Number Quantification. Anal. Chem. 84, 1003–1011 (2012). 22122760 10.1021/ac202578x PMC3260738

[b18] Hindson B. J. . High-throughput droplet digital PCR system for absolute quantitation of DNA copy number. Anal. Chem. 83, 8604–8610 (2011). 22035192 10.1021/ac202028g PMC3216358

[b19] Mazutis L. . Droplet-based microfluidic systems for high-throughput single DNA molecule isothermal amplification and analysis. Anal. Chem. 81, 4813–4821 (2009). 19518143 10.1021/ac900403z

[b20] Devonshire A. S. . Highly reproducible absolute quantification of Mycobacterium tuberculosis complex by digital PCR. Anal. Chem. 87, 3706–3713 (2015). 25646934 10.1021/ac5041617

[b21] Sousa F., Freitas S., Azzoni A. R., Prazeres D. & Queiroz J. Selective purification of SC DNA from cell lysates with single agarose chromatography step. Biot. Echnol. Biochem. 45, 131–140 (2006). 10.1042/BA20060082 16813568

[b22] Pillai V. B., Hellerstein M., Yu T., Amara R. R. & Robinson H. L. Comparative studies on *in vitro* expression and *in vivo* immunogenicity of supercoiled and open circular forms of plasmid DNA vaccines. Vaccine 26, 1136–1141 (2008). 18242791 10.1016/j.vaccine.2007.10.023 PMC2692637

[b23] CBER Draft Guidance of Considerations for Plasmid DNA Vaccines for Infectious Disease Indications. Biotechnology Law Report 24, 304–311 (2005).

[b24] CFDA Technical Guideline for Pre–Clinical Research of Preventive DNA Vaccines.

[b25] Urthaler J., Buchinger W. & Necina R. Improved downstream process for the production of plasmid DNA for gene therapy. Acta Biochim Pol. 52, 703–711 (2005). 16175245

[b26] Park S. Report of CCQM P154. CCQM BAWG Report (2015).

[b27] Burke D. G. . Digital polymerase chain reaction measured pUC19 marker as calibrant for HPLC measurement of DNA quantity. Anal. Chem. 85, 1657–1664 (2013). 23215355 10.1021/ac302925f

[b28] Huggett J. F. . The Digital MIQE Guidelines: Minimum Information for Publication of Quantitative Digital PCR Experiments. Clin. Chem. 59, 892–902 (2013). 23570709 10.1373/clinchem.2013.206375

[b29] Weaver S. . Taking qPCR to a higher level: analysis of CNV reveals the power of high throughput qPCR to enhance quantitative resolution. Methods 50, 271–276 (2010). 20079846 10.1016/j.ymeth.2010.01.003

