# Supplementary material for: Accurate quantification of supercoiled DNA by digital PCR
Source: Sci Rep. 2016 Apr 11;6:24230. doi: 10.1038/srep24230 (PMC4827127; doi:10.1038/srep24230)
Supplement: Supplementary Information [file srep24230-s1.doc]

**Accurate Quantification of Supercoiled DNA by Digital PCR**

Authors: Lianhua Dong^1^*, Hee-Bong Yoo^2,3^, Jing Wang^1^, Sang-Ryoul Park^2,3^

^1^ National Institute of Metrology, Beijing, 100013, P.R.China,

^2^ Korea Research Institute of Standards and Science, Daejeon, Korea

^3^ University of Science and Technology, Daejeon, Korea

**Supplementary material**

**EcoR1 digestion**

**
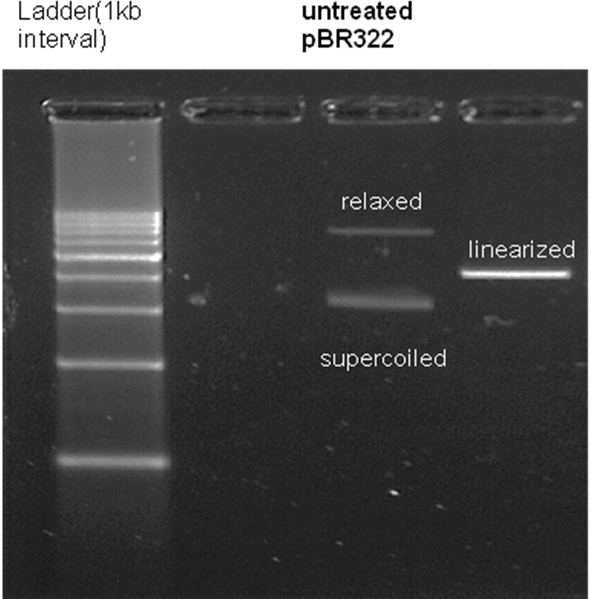

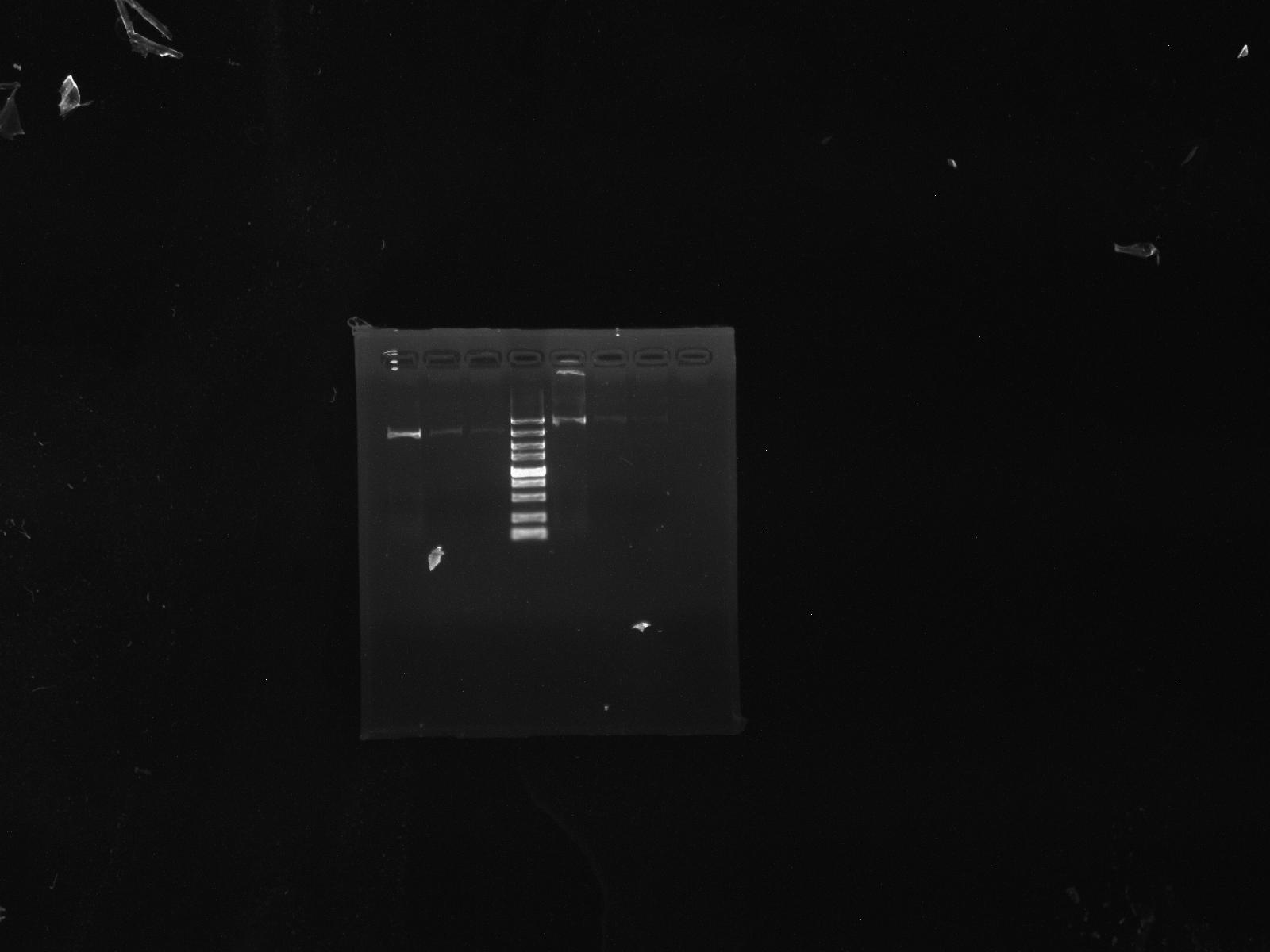
**

1 2 3 4 5 6 7 8

**A**

**B**

**Figure S1. Electrophoresis of supercoiled plasmid and its linearized plasmid digested with EcoR1 (A, pBR322 plasmid, B, pNIM-002 plasmid, B1-B3, supercoiled pNIM-002 plasmid DNA at three different concentration; B4, marker, B5-B7, linearized pNIM-002 plasmid DNA at three different concentration; B8, negative control).**

linear

a

supercoil

linear

supercoil

b

supercoil

linear

c

Figure S2. PCR amplification curves of linear and supercoiled pNIM-001 plasmid with (a) Gene Expression master mix (GE), (b) Environment master mix (EN) and (c) 16S DNA Free master mix (DF).

linear

supercoil

c

a

linear

supercoil

b

supercoil

linear

Figure S3. PCR amplification curves of linear and supercoiled pNIM-002 plasmid with (a) Gene Expression master mix (GE), (b) Environment master mix (EN) and (c) 16S DNA Free master mix (DF).


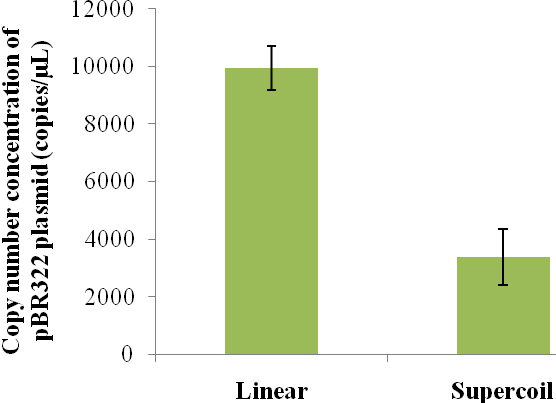


*

a


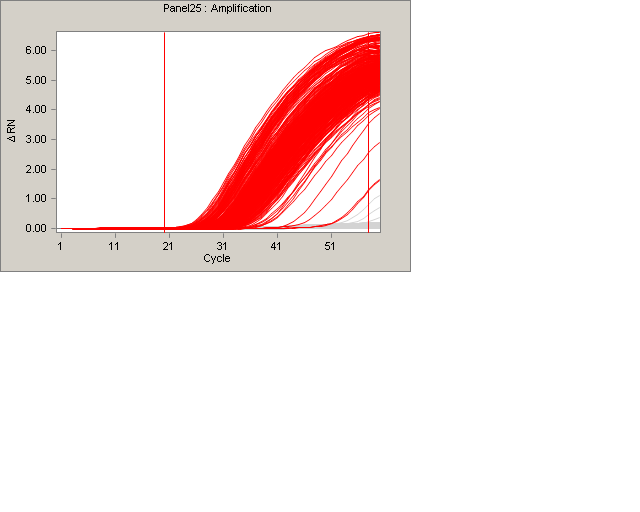

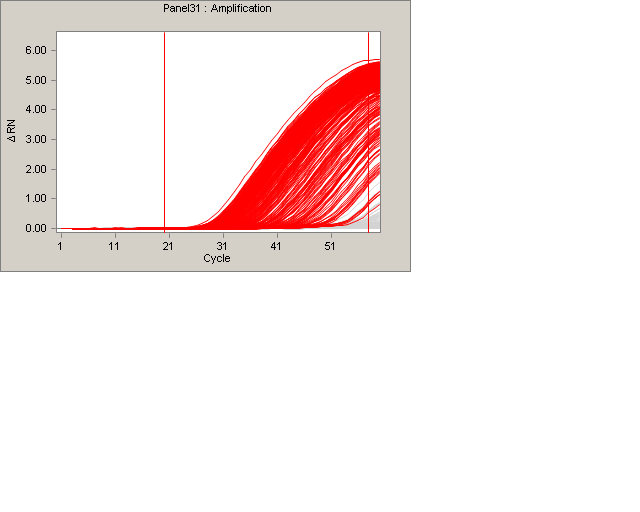


b

c

Figure S4. Comparison of digital PCR quantification with gene expression master mix (GE) for linearlized and supercoiled pBR322 plasmid (comparison sample B) using Assay I (statistically significant for Linear and Supercoil DNA, *p* =0.0008) (a, quantification result of linear and supercoiled plasmid; b, amplification curve of the linear plasmid; b, amplification curve of the supercoild plasmid).


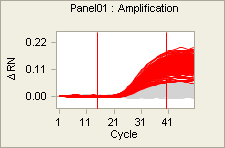

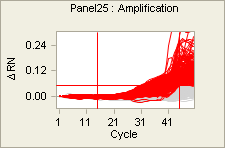


Figure S5. dPCR amplification curve of Assay I (Panel01) and Assay II (Panel25) labeled with FAM fluorophor.


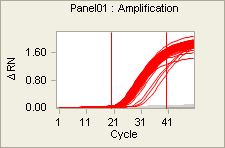

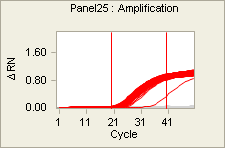


Figure S6. dPCR amplification curve of Assay I (Panel01) and Assay II (Panel25) labeled with HEX fluorophor.


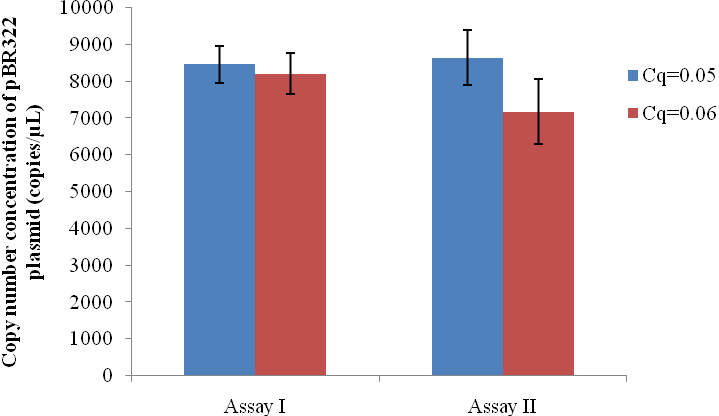


*

Figure S7. Comparison of plasmid quantification result at different dPCR Cq threshold by dPCR Assay I and Assay II labeled with FAM fluorophor. Plasmid DNA was the linearized pBR322 used for CCQM P154 (*,significant difference, *p*=0.0002).


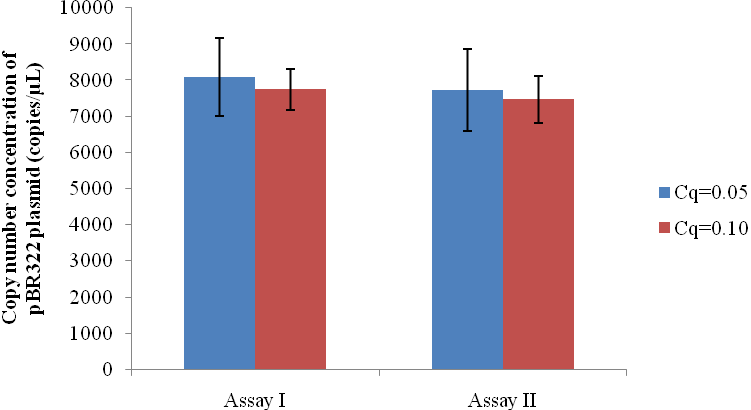


Figure S8. Comparison of plasmid quantification result at different dPCR Cq threshold by dPCR Assay I and Assay II labeled with HEX fluorophor. Plasmid DNA was the linearized pBR322 used for CCQM P154 (statistically insignificant for Assay I and Assay II, *p >0.05*).


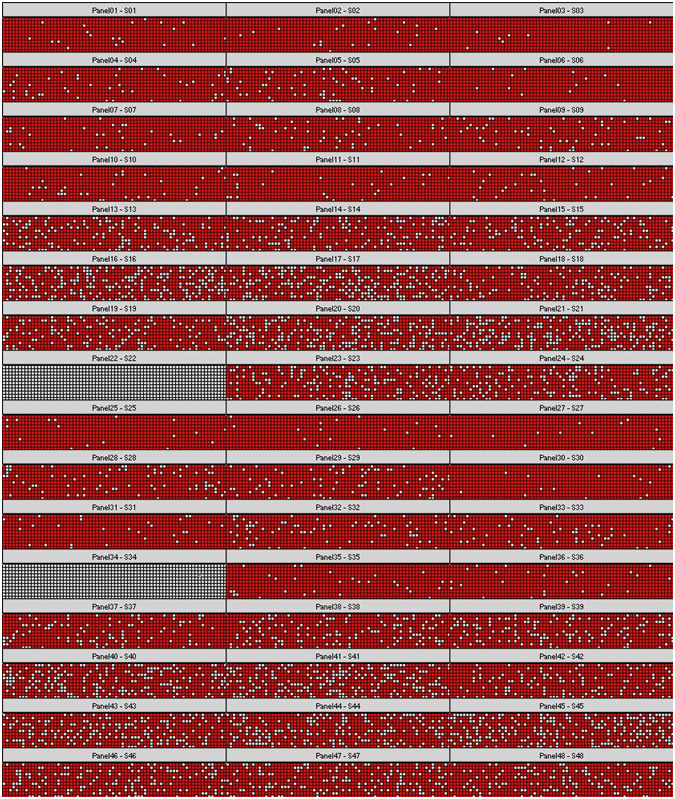


Figure S9. Result of dPCR hit map for sample concentration optimization with assay 3 and assay 4. Panel 01-24, HEX labeled Assay I and; panel25-48, HEX labeled Assay II. Panel01-12, no dilution of sample A, B and C quantified by Assay I. Panel22, NTC of Assay I. Panel13-24, two times dilution of sample A, B and C quantified by Assay I. Panel25-36, no dilution of sample A, B and C quantified by Assay II; Panel 34, NTC of Assay II; Panel37-48, two times dilution of sample A, B and C quantified by Assay II.


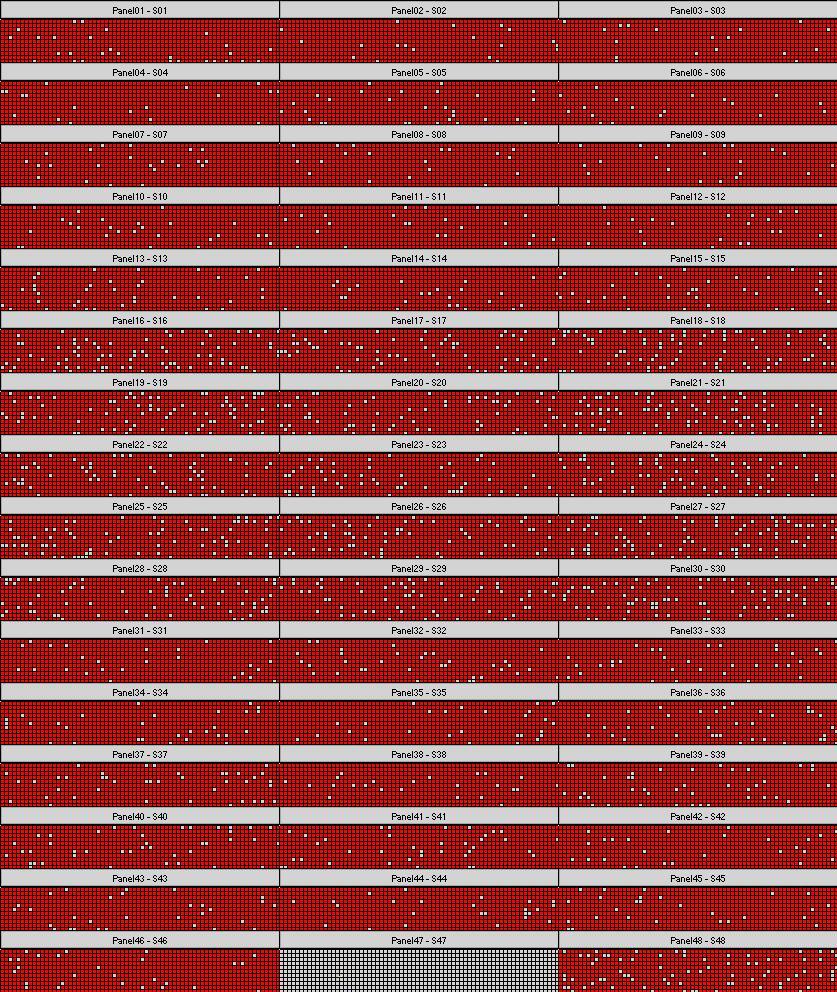


Figure S10. dPCR hit map result for sample quantification using HEX labeled Assay I. Panel 01-45, no dilution of sample A2, A3, A4, B2, B3, B4, C2, C3 and C4 with five replicates. Panel 46, C2; Panel 47, NTC; Panel 48, B2.


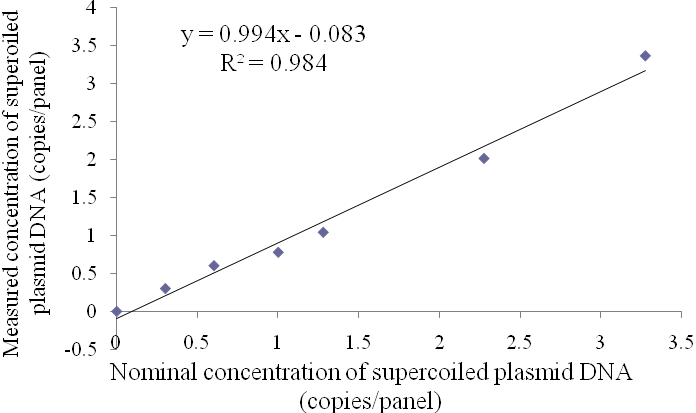


Figure S11. The linear relationship between nominal concentration and measured concentration of supercoiled pBR322 DNA (the value showed in x and y axis are the logarithm of the copies per panel).

Table S1 Primer and probe sequence for quantifying pBR322 plasmid DNA by qPCR

| Assay | Primer/  probe | Position | Sequence (5’-3’) | Concentration  (nM) | Amplicon  (bp) |
| --- | --- | --- | --- | --- | --- |
| Assay I | 3-F | pBR322-2040F | CGACCACGCTGATGAGCTT | 400 | 66 |
|  | 3-R | pBR322-2105R | CAGAGGTTTTCACCGTCATCAC | 400 |  |
|  | 3-P | pBR322-2060P | FAM-ACCGCAGCTGCCTCGCGC-BHQ1/  HEX-ACCGCAGCTGCCTCGCGC-BHQ1 | 300 |  |
| Assay II | 4-F | pBR322-1874F | TTACCCCCATGAACAGAAATCC | 400 | 73 |
|  | 4-R | pBR322-1946R | ATGTTAAGGGCGGTTTTTTCC | 400 |  |
|  | 4-P | pBR322-1897P | FAM-CCTTACACGGAGGCATCAGTGACCAAA- BHQ1/  HEX-CCTTACACGGAGGCATCAGTGACCAAA- BHQ1 | 200 |  |

Table S2 Sequence of the primer and probe for qPCR assay used for pNIM-001 and pNIM-002 plasmid DNA

| Assay | Primer/  probe | Sequence (5’-3’) | Concentration  (nM) | Amplicon  (bp) |
| --- | --- | --- | --- | --- |
| NK603 for  pNIM-001 | NK603-F | ATGAATGACCTCGAGTAAGCTTGTTAA | 400 | 108 |
|  | NK603-R | AAGAGATAACAGGATCCACTCAAACACT | 400 |  |
|  | NK603-P | FAM-TGGTACCACGCGACAGACTTCCACTC-BHQ1 | 200 |  |
| BT11 for pNIM-002 | BT11-F | GCGGAACCCCTATTTGTTTA | 400 | 93 |
|  | BT11-R | CAAGAAATGGTCTCCACCAAA | 400 |  |
|  | BT11-P | FAM-TATCCGCTCATGGAGGGATTCTTGGA-BHQ1 | 200 |  |

Table S3 PCR reaction components for qPCR assay amplifying plasmid of pBR322, pNIM-001 and pNIM-002.

| **Assay 3 for pBR322** | | | | | | | | |
| --- | --- | --- | --- | --- | --- | --- | --- | --- |
| Regent | per reaction | 30 reactions | Regent | per reaction | 30 reactions | Regent | per reaction | 30 reactions |
| GE | 12.5 | 375 | EN | 12.5 | 375 | DF | 10 | 300 |
| 3-F* | 1 | 30 | 3-F | 1 | 30 | taq E | 0.8 | 24 |
| 3-R* | 1 | 30 | 3-R | 1 | 30 | 3-F | 1 | 30 |
| 3-P* | 0.75 | 22.5 | 3-P | 0.75 | 22.5 | 3-R | 1 | 30 |
| 1×TE_0.1_ | 4.75 | 142.5 | 1×TE_0.1_ | 4.75 | 142.5 | 3-P | 0.75 | 22.5 |
| DNA | 5 | - | DNA | 5 | - | 1×TE_0.1_ | 6.45 | 193.5 |
|  |  |  |  |  |  | DNA | 5 | - |
| Total | 25 | - | Total | 25 | - | Total | 25 | - |
| **Assay 4 for pBR322** | | | | | | | | |
| Regent | per reaction | 30 reactions | Regent | per reaction | 30 reactions | Regent | per reaction | 30 reactions |
| GE | 12.5 | 375 | EN | 12.5 | 375 | DF | 10 | 300 |
| 4-F* | 1 | 30 | 4-F | 1 | 30 | taq E | 0.8 | 24 |
| 4-R* | 1 | 30 | 4-R | 1 | 30 | 4-F | 1 | 30 |
| 4-P* | 0.5 | 15 | 4-P | 0.5 | 15 | 4-R | 1 | 30 |
| 1×TE0.1 | 5 | 150 | 1×TE0.1 | 5 | 150 | 4-P | 0.5 | 15 |
| DNA | 5 | - | DNA | 5 | - | 1×TE0.1 | 6.7 | 201 |
|  |  |  |  |  |  | DNA | 5 | - |
| Total | 25 | - | Total | 25 | - | Total | 25 | - |
| **Assay NK603 for pNIM-001** | | | | | | | | |
| Regent | per reaction | 30 reactions | Regent | per reaction | 30 reactions | Regent | per reaction | 30 reactions |
| GE | 12.5 | 375 | EN | 12.5 | 375 | DF | 10 | 300 |
| NK603-F* | 1 | 30 | NK603-F | 1 | 30 | taq E | 0.8 | 24 |
| NK603-R* | 1 | 30 | NK603-R | 1 | 30 | NK603-F | 1 | 30 |
| NK603-P* | 0.5 | 15 | NK603-P | 0.5 | 15 | NK603-R | 1 | 30 |
| 1×TE0.1 | 5 | 150 | 1×TE0.1 | 5 | 150 | NK603-P | 0.5 | 15 |
| DNA | 5 | - | DNA | 5 | - | 1×TE0.1 | 6.7 | 201 |
|  |  |  |  |  |  | DNA | 5 | - |
| Total | 25 | - | Total | 25 | - | Total | 25 | - |
| **Assay BT11 for pNIM-002** | | | | | | | | |
| Regent | per tube | 30 reactions | Regent | per tube | 30 reactions | Regent | per tube | 30 reactions |
| GE | 12.5 | 375 | EN | 12.5 | 375 | DF | 10 | 300 |
| BT11-F* | 1 | 30 | BT11-F | 1 | 30 | taq E | 0.8 | 24 |
| BT11-R* | 1 | 30 | BT11-R | 1 | 30 | BT11-F | 1 | 30 |
| BT11-P* | 0.5 | 15 | BT11-P | 0.5 | 15 | BT11-R | 1 | 30 |
| 1×TE0.1 | 5 | 150 | 1×TE0.1 | 5 | 150 | BT11-P | 0.5 | 15 |
| DNA | 5 | - | DNA | 5 | - | 1×TE0.1 | 6.7 | 201 |
|  |  |  |  |  |  | DNA | 5 | - |
| Total | 25 | - | Total | 25 | - | Total | 25 | - |

GE, Gene Expression master mix; EN, Environmental master mix; DF, 16S DNA free master mix.

* stock concentration: 10µM.

Table S4 dPCR component and number of reactions for concentration optimization and assay comparison

| Assay I | | | Assay II | | |
| --- | --- | --- | --- | --- | --- |
| Component | Per reaction | 30 reactions | Component | Per reaction | 30 reactions |
| DF* | 2 | 60 | DF | 2 | 60 |
| ROX | 0.1 | 3 | ROX | 0.1 | 3 |
| DNA free taq E | 0.16 | 4.8 | DNA free taq E | 0.16 | 4.8 |
| 20×GE loading | 0.25 | 7.5 | 20×GE loading | 0.25 | 7.5 |
| 3 F | 0.2 | 6 | 4 F | 0.2 | 6 |
| 3 R | 0.2 | 6 | 4 R | 0.2 | 6 |
| 3P-HEX | **0.15** | 4.5 | 4P-HEX | **0.1** | 3 |
| 1×TE_0.1_ | 0.94 | 28.2 | 1× TE_0.1_ | 0.99 | 29.7 |
| DNA | 1 | - | DNA | 1 | - |
| Total with DNA | 5 | 120 | Total with DNA | 5 | 120 |

*DF, 16S DNA free master mix

Table S5 Result of concentration optimization and assay comparison by dPCR

| Assay | ID | Sample | Positive count | Copies/  panel | Mean copies/partition | Stocked concentration (Copies/mg) |
| --- | --- | --- | --- | --- | --- | --- |
| Assay I | 1 | A1 | 750 | 2831 | 3.65 | 20173 |
|  | 2 | A1 | 750 | 2831 | 3.65 | 20173 |
|  | 3 | A1 | 761 | 3477 | 4.45 | 24777 |
|  | 6 | A1 | 757 | 3176 | 4.08 | 22632 |
|  | 4 | B1 | 726 | 2212 | 2.86 | 16471 |
|  | 5 | B1 | 709 | 1958 | 2.54 | 14580 |
|  | 8 | B1 | 719 | 2097 | 2.71 | 15615 |
|  | 9 | B1 | 713 | 2011 | 2.60 | 14974 |
|  | 7 | C1 | 738 | 2461 | 3.18 | 18324 |
|  | 10 | C1 | 734 | 2369 | 3.06 | 17639 |
|  | 11 | C1 | 745 | 2655 | 3.43 | 19768 |
|  | 12 | C1 | 738 | 2461 | 3.18 | 18324 |
|  | 13 | A1X2 | 630 | 1315 | 1.70 | 19421 |
|  | 14 | A1X2 | 634 | 1337 | 1.73 | 19746 |
|  | 15 | A1X2 | 643 | 1390 | 1.80 | 20528 |
|  | 18 | A1X2 | 640 | 1372 | 1.78 | 20262 |
|  | 16 | B1X2 | 538 | 925 | 1.20 | 14035 |
|  | 17 | B1X2 | 539 | 928 | 1.20 | 14080 |
|  | 20 | B1X2 | 548 | 959 | 1.24 | 14551 |
|  | 21 | B1X2 | 542 | 938 | 1.22 | 14232 |
|  | 22 | NTC | 0 | 0 | 0.00 | - |
|  | 23 | C1X2 | 602 | 1174 | 1.52 | 17275 |
|  | 24 | C1X2 | 618 | 1251 | 1.62 | 18408 |
|  | 19 | C1X2 | 631 | 1320 | 1.71 | 19423 |
| Assay II | 25 | A1 | 753 | 2961 | 3.81 | 21253 |
|  | 26 | A1 | 753 | 2961 | 3.81 | 21253 |
|  | 27 | A1 | 758 | 3241 | 4.16 | 23262 |
|  | 30 | A1 | 755 | 3061 | 3.94 | 21970 |
|  | 28 | B1 | 695 | 1798 | 2.33 | 13501 |
|  | 29 | B1 | 692 | 1768 | 2.29 | 13275 |
|  | 32 | B1 | 697 | 1819 | 2.36 | 13658 |
|  | 33 | B1 | 714 | 2025 | 2.62 | 15205 |
|  | 34 | NTC | 0 | 0 | 0.00 | - |
|  | 31 | C1 | 741 | 2538 | 3.28 | 18057 |
|  | 35 | C1 | 741 | 2538 | 3.28 | 18057 |
|  | 36 | C1 | 743 | 2595 | 3.35 | 18462 |
|  | 37 | A1X2 | 673 | 1599 | 2.07 | 23205 |
|  | 38 | A1X2 | 642 | 1384 | 1.79 | 20085 |
|  | 39 | A1X2 | 636 | 1349 | 1.75 | 19577 |
|  | 42 | A1X2 | 652 | 1447 | 1.88 | 20999 |
|  | 40 | B1X2 | 563 | 1013 | 1.31 | 14696 |
|  | 41 | B1X2 | 546 | 952 | 1.23 | 13811 |
|  | 44 | B1X2 | 578 | 1071 | 1.39 | 15538 |
|  | 45 | B1X2 | 535 | 915 | 1.19 | 13274 |
|  | 43 | C1X2 | 586 | 1104 | 1.43 | 15972 |
|  | 46 | C1X2 | 617 | 1246 | 1.62 | 18026 |
|  | 47 | C1X2 | 620 | 1262 | 1.64 | 18258 |
|  | 48 | C1X2 | 641 | 1378 | 1.79 | 19936 |

Table S6 Result of the quantification of all unknown sample

| ID | Sample | Positive count | Copies/  panel | Mean copies/partition | Stocked concentration (Copies/mg) | Average of stocked concentration (copies/mg) |
| --- | --- | --- | --- | --- | --- | --- |
| 1 | A2 | 742 | 2566 | 3.31 | 20790 | 22371 |
| 2 |  | 751 | 2872 | 3.70 | 23269 |  |
| 3 |  | 752 | 2915 | 3.76 | 23617 |  |
| 4 |  | 752 | 2915 | 3.76 | 23617 |  |
| 5 |  | 741 | 2538 | 3.28 | 20563 |  |
| 6 | A3 | 752 | 2915 | 3.76 | 22558 | 21665 |
| 7 |  | 748 | 2756 | 3.56 | 21328 |  |
| 8 |  | 751 | 2872 | 3.70 | 22225 |  |
| 9 |  | 750 | 2831 | 3.65 | 21908 |  |
| 10 |  | 744 | 2624 | 3.39 | 20306 |  |
| 11 | A4 | 747 | 2721 | 3.51 | 20400 | 19455 |
| 12 |  | 749 | 2793 | 3.60 | 20939 |  |
| 13 |  | 734 | 2369 | 3.06 | 17761 |  |
| 14 |  | 745 | 2655 | 3.43 | 19905 |  |
| 15 |  | 737 | 2437 | 3.15 | 18270 |  |
| 16 | B2 | 694 | 1788 | 2.32 | 14003 | 14504 |
| 17 |  | 709 | 1958 | 2.54 | 15335 |  |
| 18 |  | 693 | 1778 | 2.30 | 13925 |  |
| 19 |  | 696 | 1808 | 2.34 | 14160 |  |
| 20 |  | 708 | 1946 | 2.52 | 15241 |  |
| 48 |  | 693 | 1778 | 2.30 | 14358 |  |
| 21 | B3 | 688 | 1729 | 2.24 | 13504 | 14698 |
| 22 |  | 709 | 1958 | 2.54 | 15293 |  |
| 23 |  | 715 | 2039 | 2.64 | 15926 |  |
| 24 |  | 703 | 1885 | 2.44 | 14723 |  |
| 25 |  | 695 | 1798 | 2.33 | 14043 |  |
| 26 | B4 | 708 | 1946 | 2.52 | 14926 | 14458 |
| 27 |  | 692 | 1768 | 2.29 | 13560 |  |
| 28 |  | 706 | 1921 | 2.49 | 14734 |  |
| 29 |  | 710 | 1971 | 2.55 | 15117 |  |
| 30 |  | 697 | 1819 | 2.36 | 13951 |  |
| 31 | C2 | 744 | 2624 | 3.39 | 20055 | 19275 |
| 32 |  | 734 | 2369 | 3.06 | 18106 |  |
| 33 |  | 737 | 2437 | 3.15 | 18625 |  |
| 34 |  | 740 | 2512 | 3.25 | 19199 |  |
| 35 |  | 743 | 2595 | 3.35 | 19833 |  |
| 46 |  | 743 | 2595 | 3.35 | 19833 |  |
| 36 | C3 | 733 | 2348 | 3.04 | 17829 | 17978 |
| 37 |  | 729 | 2267 | 2.93 | 17214 |  |
| 38 |  | 746 | 2687 | 3.47 | 20404 |  |
| 39 |  | 730 | 2287 | 2.96 | 17366 |  |
| 40 |  | 728 | 2249 | 2.91 | 17078 |  |
| 41 | C4 | 732 | 2327 | 3.01 | 16368 | 17884 |
| 42 |  | 744 | 2624 | 3.39 | 18457 |  |
| 43 |  | 740 | 2512 | 3.25 | 17669 |  |
| 44 |  | 745 | 2655 | 3.43 | 18675 |  |
| 45 |  | 743 | 2595 | 3.35 | 18253 |  |

Table S7 The supercoiled DNA concentration (copies/µL) in each dilution and the measured copies per panel by dPCR

| Supercoiled pBR322 | Concentration* (copies /µL) | Nominal concentration (copies/panel) | Measured concentration by dPCR (copies/panel) |
| --- | --- | --- | --- |
| SD1 | 13984 | 1878 | 2302 |
| SD2 | 1402 | 188 | 103 |
| SD3 | 142 | 19 | 11 |
| SD4 | 71 | 10 | 6 |
| SD5 | 30 | 4 | 4 |
| SD6 | 14 | 2 | 2 |
| SD7 | 8 | 1 | 1 |

*DNA concentration before diluted with PCR mixture.
